# Exacerbation of psychotic symptoms as clinical presentation of Wernicke encephalopathy in an Alzheimer's disease patient

**DOI:** 10.1002/jgf2.330

**Published:** 2020-05-17

**Authors:** Nozomu Uchida, Mayumi Ishida, Izumi Sato, Takao Takahashi, Daisuke Furuya, Yasuhiro Ebihara, Hiroshi Ito, Akira Yoshioka, Hideki Onishi

**Affiliations:** ^1^ Department of General Medicine Ogano Town Central Hospital Saitama Japan; ^2^ Department of Psycho‐oncology Saitama Medical University International Medical Center Saitama Japan; ^3^ Department of Pharmacoepidemiology Graduate School of Medicine and Public Health Kyoto University Kyoto Japan; ^4^ Department of Supportive Medicine Saitama Medical University International Medical Center Saitama Japan; ^5^ Department of General Medicine Saitama Medical University International Medical Center Saitama Japan; ^6^ Department of Laboratory Medicine Saitama Medical University International Medical Center Saitama Japan; ^7^ Ito Internal Medicine and Pediatric Clinic Fukuoka Japan; ^8^ Department of Medical Oncology Mitsubishi Kyoto Hospital Kyoto Japan

**Keywords:** BPSD, delirium, dementia, elderly people, thiamine deficiency (TD)

## Abstract

Although there have been recent reports of nonalcoholic thiamine deficiency (TD), no association has been reported between the exacerbation of the psychiatric symptoms of Alzheimer's disease patient and TD. An 89‐year‐old woman with dementia visited our hospital because of acute deterioration in behavioral and psychological symptoms of dementia (BPSD). Her medical history revealed a decrease in oral food intake lasting more than 2 weeks, so that TD was suspected and abnormal behavior improved significantly after thiamine administration. Thiamine deficiency should be suspected in patients with dementia who demonstrate acute deterioration in BPSD possibly related to poor oral food intake.

## 1 INTRODUCTION

Thiamine, in its biologically active form, thiamine pyrophosphate, is an essential coenzyme for glucose metabolism.^1^ However, humans cannot synthesize thiamine, so they are reliant on intake from external sources.^2^ As the store of thiamine in the body is depleted in as few as about 18 days,^3^ thiamine deficiency (TD) may occur if anorexia or similar conditions last for 2‐3 weeks.^2^, ^4^ Such TD typically leads to Wernicke encephalopathy (WE).^1^ This disorder has historically and mistakenly been described by a classic triad of signs: mental‐status changes, ataxia, and nystagmus/ophthalmoplegia.^1^ Treatment consists of the intravenous administration of thiamine, and if it is discovered and treated early, patients generally recover without complications.^1^, ^5^ However, the classical triad of symptoms show low disease specificity and not all symptoms are always present; therefore, the diagnosis of WE is often overlooked.^4^, ^6^

If the deficiency of thiamine continues, Korsakoff syndrome could eventually develop. Korsakoff syndrome is characterized by anterograde amnesia and fabricated or distorted speech, causing severe impairment in daily life and sometimes leading to death.^7^

However, the diagnosis of TD in a clinical setting is rare unless the attending physician specifically measures thiamine levels.^7^ Therefore, the actual status regarding TD is not well understood in outpatients.

Here, we report the case of a patient who demonstrated deterioration in BPSD, such as forgetfulness, day‐night reversal, night wandering, and hallucinations, apparently related to a decrease in oral food intake lasting more than 2 weeks. We interpreted the acute deterioration in BPSD in the patient as a clinical pattern caused by WE, since the symptoms improved significantly after thiamine administration.

To determine the etiology of the delirium, the Francis criteria^8^ were employed to standardize judgments. On the basis of clinical assessment and a medical chart review, the potential cause was categorized as: (a) definite: If it was temporally related, there was laboratory confirmation, the patient improved with treatment or cessation of exposure to the offending agent, and there was no other cause present; or (b) probable: If all the previous criteria were met but another main cause was present, or laboratory confirmation was not obtained.

## 2 CASE REPORT

An 89‐year‐old woman with a history of Alzheimer's disease was referred to our hospital for assessment by her physician because of acute deterioration in BPSD. In terms of activities of daily life, she was able to walk independently and was able to eat on her own when food was provided. Her most recent Revised Hasegawa Dementia Scale (HDS‐R) score was nine points. She made use of a daycare service during the day. According to a report by a staff member at the daycare facility, her forgetfulness had worsened over the previous week, and she had experienced day‐night reversal, night wandering, and the illusion that someone was resting on her head. She had almost no water intake, and she had consumed only a few mouthfuls of food, including both staple foodstuffs and side dishes, at meals. And multivitamin supplementation had not been commenced.

The findings at the first consultation were as follows. The patient was 127.5 cm in height and 34.3 kg in weight, with a BMI of 21.1. Her blood pressure was 127/69 mm Hg, heart rate 68 bpm, and body temperature 36.6°C.

Although conversation was possible, she could not tell the time or date, and she appeared disoriented. Furthermore, although she answered questions, she could not give correct answers as she did not understand the questions. Examination of her conjunctiva showed no sign of anemia, chest examination showed no murmur or abnormal breathing, and her abdomen was flat, soft, and without areas of tenderness. Her laboratory findings were as follows: white blood cell count, 6530/µL; red blood cell count, 417 × 10^4^/µL; hemoglobin, 9.9 g/dL; hematocrit, 29.4%; platelet count, 33.4 × 10^4^/µL; aspartate aminotransferase, 28 IU/L; alanine aminotransferase, 13 IU/L; alkaline phosphatase, 209 IU/L; lactate dehydrogenase (LDH), 327 IU/L; gamma‐glutamyl transpeptidase, 8 IU/L; blood urea nitrogen, 26.3 mg/dL; creatinine, 1.2 mg/dL; sodium (Na), 140 mEq/L; potassium (K), 2.5 mEq/L; and chlorine (Cl), 86 mEq/L. Chest x‐rays revealed no obvious congestion, cardiac enlargement, or infiltrative shadows. CT scans of the head showed atrophy of the frontal and temporal lobes and the hippocampus, and her electrocardiogram revealed mild ST depression in leads I, II, aVF, and V2‐6 (possibly because of hypokalemia). Further interviews with a staff member at the daycare facility revealed that her oral food intake had been decreased for 2 months.

The worsening of her psychiatric symptoms within a period of only 2 weeks was considered to indicate delirium rather than the progression of Alzheimer's disease. In addition, TD was suspected from the fact that her dietary intake had been decreased for over 2 months and thiamine is only stored in the body for about 18 days.^3^ Therefore, as she satisfied 2 out of 4 of Caine's WE diagnostic criteria—eye signs, cerebellar signs, mild memory impairment or confusion, and signs of malnutrition^9^—she was suspected of TD.

Thiamine disulfide (50 mg) was administered intravenously once a day for 3 days. In addition, we administered an infusion of maintenance fluid at 500 mL a day for 3 days because of her decreased water intake. Her symptoms such as day‐night reversal, night wandering, and hallucinations disappeared a few days later. Several days after blood sampling, her serum thiamine level was found to be abnormally reduced to 19.4 (normal range: 21.3‐81.9 ng/mL). Thereafter, she returned to her regular life.

The clinical findings, effective alleviation of delirious symptoms after thiamine and the administration of maintenance fluid infusion, low thiamine intake from food, and low level of thiamine in the serum fulfilled the Francis criteria^8^ for delirium induced by thiamine deficiency.

Over the following 9 months, the patient showed no recurrence of delirium. The VB1 blood concentration measured at that time was 37.0 ng/mL.

## 3 DISCUSSION

In this report, we documented a case of TD likely related to deterioration in BPSD in an Alzheimer's disease patient.

To date, the reason why thiamine has not been considered to be involved in the deterioration in BPSD in a general clinical setting may be that TD is not often suspected.^7^ In addition, the symptoms of TD are various and can be frequently overlooked as there are no specific symptoms.^4^, ^6^, ^10^ In other words, when patients with dementia initially experience a deterioration in BPSD, it may not be thought to be related to the symptoms of TD. Furthermore, as multivitamin preparations are blindly mixed in infusions for the treatment of patients with poor oral food intake,^11^ it may be that WE is prevented before deterioration in BPSD becomes apparent in the majority of patients with dementia.

The clue to the diagnosis of TD in this patient was the decline in oral food intake lasting for about 2 months that was identified from a review of her records and reports from the staff of her regular daycare service. Thiamine stores in the body are depleted in as few as 18 days.^3^ As this patient could not talk to us about her appetite, careful monitoring of appetite by the staff of daycare facilities is advisable.

As a limitation to this case, guidelines for the treatment of TD recommend the use of intravenous thiamine of 200 mg or more.^6^ However, there have been reports of cases in which symptoms were improved by oral administration.^12^, ^13^ In this case, we administered 50 mg of thiamine as an initial response, but later found the patient to be suffering from TD. Therefore, it is desirable to treat similar cases in the future according to the guidelines.

It has been reported that about half of elderly men and 39% of elderly women do not have an appropriate thiamine intake.^14^ In addition, it has been reported that many patients with Alzheimer's disease have TD,^15^ and those presenting with dementia and delirium have lower average thiamine blood levels than those without such conditions.^16^ Therefore, the fact that TD can easily occur when oral food intake is decreased in elderly people with dementia should not be neglected. In our aging society, the number of elderly people with dementia is expected to increase; therefore, it is very important in our daily work that we remain aware of the fact that TD may cause deterioration in BPSD. Continued research is expected to help clarify both the existence of thiamine‐deficient patients and the factors related to its onset, thereby allowing the early detection of potential thiamine‐deficient patients and contributing to the prevention of WE and Korsakoff syndrome.

## 4 CONCLUSION

The elderly or those with dementia who have poor oral food intake for more than 2 weeks should be examined for possible TD. In the future, we will conduct further research to investigate the rate and determinants of TD in this population.

## ACKNOWLEDGEMENT

None

## CONFLICT OF INTEREST

The authors have stated explicitly that there are no conflicts of interest in connection with this article.

## CONSENT FOR PUBLICATION

Written informed consent was obtained from the patient and patient's family for publication of this case report.

## References

[1] Sechi G , Serra A . Wernicke's encephalopathy: new clinical settings and recent advances in diagnosis and management. Lancet Neurol. 2007;6(5):442–55. 17434099 10.1016/S1474-4422(07)70104-7

[2] Sechi G , Sechi E , Fois C , Kumar N . Advances in clinical determinants and neurological manifestations of B vitamin deficiency in adults. Nutr Rev. 2016;74(5):281–300. 27034475 10.1093/nutrit/nuv107

[3] MacLean LD , Rhode BM , Shizgal HM . Nutrition following gastric operations for morbid obesity. Ann Surg. 1983;198(3):347–55. 6615057 10.1097/00000658-198309000-00011 PMC1353306

[4] Onishi H , Ishida M , Toyama H , Tanahashi I , Ikebuchi K , Taji Y , et al. Early detection and successful treatment of Wernicke encephalopathy in a patient with advanced carcinoma of the external genitalia during chemotherapy. Palliat Support Care. 2016;14(3):302–6. 26653343 10.1017/S1478951515000875

[5] Isenberg‐Grzeda E , Hsu AJ , Hatzoglou V , Nelso C , Breitbart W . Palliative treatment of thiamine‐related encephalopathy (Wernicke's encephalopathy) in cancer: a case series and review of the literature. Palliat Support Care. 2015;13(5):1241–9. 25339378 10.1017/S1478951514001163 PMC4982657

[6] Isenberg‐Grzeda E , Kutner HE , Nicolson SE . Wernicke‐Korsakoff‐syndrome: under‐recognized and under‐treated. Psychosomatics. 2012;53(6):507–16. 23157990 10.1016/j.psym.2012.04.008

[7] Isenberg‐Grzeda E , Rahane S , DeRosa AP , Ellis J , Nicolson SE . Wernicke‐Korsakoff syndrome in patients with cancer: a systematic review. Lancet Oncol. 2016;17(4):e142–e148. 27300674 10.1016/S1470-2045(16)00037-1

[8] Francis J , Martin D , Kapoor WN . A prospective study of delirium in hospitalized elderly. JAMA. 1990;263(8):1097–101. 2299782

[9] Caine D , Halliday GM , Kril JJ , Harper CG . Operational criteria for the classification of chronic alcoholics: identification of Wernicke's encephalopathy. J Neurol Neurosurg Psychiatry. 1997;62(1):51–60. 9010400 10.1136/jnnp.62.1.51 PMC486695

[10] Onishi H , Ishida M , Tanahashi I , Takahashi T , Taji Y , Ikebuchi K , et al. Wernicke encephalopathy without delirium in patients with cancer. Palliat Support Care. 2018;16(1):118–21. 28464962 10.1017/S1478951517000360

[11] Shikata E , Mizutani T , Kokubun Y , Takasu T . ‘Iatrogenic’ Wernicke's encephalopathy in Japan. Eur Neurol. 2000;44(3):156–61. 11053964 10.1159/000008226

[12] Onishi H , Ishida M , Tanahashi I , Takahashi T , Ikebuchi K , Taji Y , et al. Early detection and successful treatment of Wernicke's encephalopathy in outpatients without the complete classic triad of symptoms who attended a psycho‐oncology clinic. Palliat Support Care. 2018;16(5):633–6. 29478423 10.1017/S1478951518000032

[13] Wilkinson TJ , Hanger HC , Elmslie J , George PM , Sainsbury R . The response to treatment of subclinical thiamine deficiency in the elderly. Am J Clin Nutr. 1997;66(4):925–8. 9322569 10.1093/ajcn/66.4.925

[14] ter Borg S , Verlaan S , Hemsworth J , Mijnarends DM , Schols JM , Luiking YC , et al. Micronutrient intakes and potential inadequacies of community‐dwelling older adults: a systematic review. Br J Nutr. 2015;113(8):1195–206. 25822905 10.1017/S0007114515000203 PMC4531469

[15] Gibson GE , Hirsch JA , Fonzetti P , Jordan BD , Cirio RT , Elder J . Vitamin B1 (thiamine) and dementia. Ann N Y Acad Sci. 2016;1367(1):21–30. 26971083 10.1111/nyas.13031 PMC4846521

[16] Pourhassan M , Angersbach B , Lueg G , Klimek CN , Wirth R . Blood thiamine level and cognitive function in older hospitalized patients. J Geriatr Psychiatry Neurol. 2018;32(2):90–6. 30572755 10.1177/0891988718819862

